# Human Umbilical Cord Blood-Derived Mesenchymal Stem Cells Contribute to Chondrogenesis in Coculture with Chondrocytes

**DOI:** 10.1155/2016/3827057

**Published:** 2016-06-30

**Authors:** Xingfu Li, Li Duan, Yujie Liang, Weimin Zhu, Jianyi Xiong, Daping Wang

**Affiliations:** ^1^Shenzhen Key Laboratory of Tissue Engineering, Shenzhen Second People's Hospital (The First Hospital Affiliated to Shenzhen University), Shenzhen, Guangdong 518035, China; ^2^Department of Orthopedics, Shenzhen Second People's Hospital (The First Hospital Affiliated to Shenzhen University), Shenzhen, Guangdong 518035, China; ^3^Department of Chemistry, The Chinese University of Hong Kong, Shatin, Hong Kong; ^4^School of Chemical Biology & Biotechnology, Peking University Shenzhen Graduate School, Shenzhen, Guangdong 518055, China

## Abstract

Human umbilical cord blood-derived mesenchymal stem cells (hUCB-MSCs) have been shown as the most potential stem cell source for articular cartilage repair. In this study, we aimed to develop a method for long-term coculture of human articular chondrocytes (hACs) and hUCB-MSCs at low density in vitro to determine if the low density of hACs could enhance the hUCB-MSC chondrogenic differentiation as well as to determine the optimal ratio of the two cell types. Also, we compared the difference between direct coculture and indirect coculture at low density. Monolayer cultures of hUCB-MSCs and hACs were investigated at different ratios, at direct cell-cell contact groups for 21 days. Compared to direct coculture, hUCB-MSCs and hACs indirect contact culture significantly increased type II collagen (COL2) and decreased type I collagen (COL1) protein expression levels. SRY-box 9 (SOX9) mRNA levels and protein expression were highest in indirect coculture. Overall, these results indicate that low density direct coculture induces fibrocartilage. However, indirect coculture in conditioned chondrocyte cell culture medium can increase expression of chondrogenic markers and induce hUCB-MSCs differentiation into mature chondrocytes. This work demonstrates that it is possible to promote chondrogenesis of hUCB-MSCs in combination with hACs, further supporting the concept of novel coculture strategies for tissue engineering.

## 1. Introduction

Repair of cartilage defects represents a significant orthopedic challenge due to the limited healing capacity of mature cartilage; therefore, the development of new tissue engineering strategies is of major importance for cartilage repair [1]. Autologous chondrocyte implantation (ACI) has long been considered the gold standard to treat cartilage defects [2]. However, use of autologous chondrocytes has disadvantages that limit potential clinical applications, including donor site morbidity and dedifferentiation of the harvested chondrocytes after* ex vivo* monolayer expansion [3]. Recent studies have shifted focus from ACI to mesenchymal stem cell (MSC) therapy, which has been shown as effective for articular cartilage repair [4–11]. Additionally, MSCs have already shown safety and efficacy in a variety of regenerative medicine clinical trials [12–14]. In particular, human umbilical cord blood-derived MSCs (hUCB-MSCs) could serve as a promising cell source for* in vivo* repair of cartilage defects due to advantages of noninvasive collection, high proliferative potential, lower immunogenicity, and chondrogenic potential* in vitro* [15–17].

However, strategies employing hUCB-MSCs for cartilage regeneration are problematic due to the low induction efficiency of hUCB-MSCs alone, in the absence of growth factors and/or gene delivery systems to signal the stem cells to undergo chondrogenesis [18, 19]. An approach that supplements an abundant stem cell source with prochondrogenic signals and cell adhesions needs to be optimized before hUCB-MSCs can be applied therapeutically. Combining progenitors with mature chondrocytes may provide a solution, as coculture of hUCB-MSCs and chondrocytes* in vitro* has previously been shown to promote hUCB-MSC chondrogenesis and inhibit MSC hypertrophy through specific chondrocyte-secreted factors [20].

It was reported that coculture of HUCB-MSCs and rabbit chondrocytes could induce the differentiation of hUCB-MSCs into human chondrocytes, and the author also obtained the more suitable seed cells ratio [21]. In their study they seeded cells at the density of 2.4 × 10^6^ cells/cm^2^. They stated that density of chondrocyte seeding is 1–3 × 10^4^ cells/cm^2^; however, this could lead chondrocytes to a fibrotic phenotype. The aim of our study was to explore whether coculture at the density less than 1–3 × 10^4^ cells/cm^2^ could induce more chondrocytes and avoid fibrosis. If low density seed cells could induce hUCB-MSCs differentiation into enough chondrocytes for cartilage tissue engineering, the cartilage extracted from patients for chondrocyte proliferation* in vitro* could be greatly lessened.

Direct coculture and indirect coculture are usually adopted in MSCs chondrogenic differentiation induced by articular chondrocytes (ACs). However, the mechanisms of interaction between ACs and MSCs in coculture have not been fully characterized. It is speculated that both physical and paracrine interactions between these two cell types are important in maintaining the chondrogenic phenotype which results in induction of hUCB-MSC chondrogenesis [22]. Chondrocytes also secrete autocrine growth factors such as transforming growth factor-*β* (TGF-*β*) and insulin-like growth factor-1 (IGF-1), and the chondrogenic factor (SOX9) can induce chondrogenic cells [23, 24].

The effect of coculture on hUCB-MSC chondrogenesis using hACs, in particular at the low density culture, was investigated in this study. Our work also compared direct cell-cell contact and indirectly coculture of hUCB-MSC and chondrocytes for improving the coculture system. In addition, the role of TGF-*β* in the coculture system was determined. Results of this study demonstrated that low density coculture model could maintain the chondrocyte phenotype and minimize donor site injury; thus it provided an alternative chondrocytes induction and proliferation system for cartilage tissue engineering.

## 2. Materials and Methods

### 2.1. Collection of hUCB and Cartilage

The collection of human umbilical cord blood (hUCB) and cartilage was approved by Shenzhen Second People's Hospital. Informed consent was obtained before the operation from all individuals included in the study. According to the institutional guidelines, hUCB units were obtained from normal full-term and preterm deliveries without complications throughout pregnancy, in a physiological saline system containing heparin anticoagulant, and were processed within 6 hr of collection. The units were stored and transported at 4°C. No complications were encountered upon hUCB collection, and none of the samples had signs of coagulation or haemolysis. Cartilage samples were obtained from donors after trauma patients, in a physiological saline system containing penicillin/streptomycin (P/S), and were processed within 6 hr of collection.

### 2.2. Isolation and Culture of hUCB-MSCs and hACs

After hUCB was diluted 1 : 1 with 100 U/mL heparin-saline, hUCB-MSCs were isolated using Ficoll-Paque density gradient centrifugation (Amersham Biosciences, Uppsala, Sweden) and resuspended in MesenGro® human mesenchymal stem cell medium (StemRD, America) that was supplemented with 10% fetal bovine serum (FBS) (Gibco, Australia) and 10 *μ*g/L basic fibroblast growth factor (bFGF) (Gibco, Australia). Mononucleated cells were seeded into T25 cell culture flasks (Nunc, USA) at 2 × 10^4^ cells/cm^2^ and cultured at 37°C in a 5% CO_2_ incubator. Five days after the cells were seeded; nonadherent cells were removed and fresh medium was added to the flasks. Medium replacement was carried out every 72 h until the cells reached an 80% confluent layer. Cells were digested with 0.25% (w/v) trypsin plus 0.02% (w/v) EDTA (HyClone, USA) and subcultured at a density of 1.0 × 10^4^ cells/cm^2^. Medium was changed twice a week. The hUCB-MSCs of passage 3 were used for chondrocyte induction [25, 26].

Cartilage specimens were collected, minced to 1 mm^3^, and digested in the chondrocyte growth medium containing 1 mg/mL collagenase type II (Worthington Biochemical Corporation) for 8 hr at 37°C in a shaker. After filtration, cells were harvested and plated at a density of 1 × 10^4^ cells/cm^2^ and subcultured in chondrocyte growth medium (DMEM-F12, 10% FBS, 10 *μ*g/L bFGF, and 0.1 mg/mL P/S). Chondrocytes of passage 2 were used for chondrocyte induction [27].

### 2.3. Identification of HUCB-MSCs

hUCB-MSCs were suspended in PBS containing 5% bovine serum albumin (Sigma-Aldrich, USA) at a concentration of 3 × 10^5^ cells/50 *μ*L and stained with CD105, CD73, CD34, and CD45 (BD Biosciences). The appropriate human isotype antibodies were used as controls. Samples were processed using a FACSCanto II flow cytometer (BD Biosciences) and analyzed with FlowJo software (Treestar) [28].

### 2.4. Chondrogenic Induction of hUCB-MSCs

hACs (P2) and hUCB-MSCs (P3) were used. For coculture with direct cell-cell contact, hACs and hUCB-MSCs were mixed directly at ratio of 1 : 1, 3 : 1, and 5 : 1 (hUCB-MSCs : hACs) [7, 9, 29, 30] and then cultured in basal medium (DMEM-F12, 10% FBS, 100 U/mL penicillin, and 0.1 mg/mL streptomycin). For indirect coculture, hUCB-MSCs and hACs were cultured with supernatants from each other [31]. For growth factor induction in monolayer culture, hUCB-MSCs were maintained in basal medium supplemented with 0.1 mM dexamethasone, 40 mg/mL L-proline, 10 *μ*g/L transforming growth factor beta-1 (TGF-*β*1, Peprotech, USA), 10 *μ*g/L insulin-like growth factor-1 (IGF-1) (Peprotech, USA), and 1% insulin transferrin selenium (ITS, Invitrogen) [32–35]. hACs and hUCB-MSCs cultured alone with basal medium were used as controls. All cells were incubated for three weeks at 37°C in a humidified atmosphere of 5% CO_2_ and the medium changed every three days. The designated groups for this study were listed in Table 1.

### 2.5. Immunofluorescent Staining of COL2

A hyaline cartilage marker protein COL2 was examined using immunofluorescence. All groups were harvested 3 weeks after seeding. The samples were fixed in 4% paraformaldehyde for 15 min at room temperature followed by incubation with PBS containing 0.2% Triton-X100 for 15 min at room temperature (RT); then cells were blocked in 5% bovine serum albumin at RT. Then cells were subsequently incubated overnight at 4°C with mouse mAb to COL2 (1 : 100, R&D Systems). Subsequently, samples were washed and incubated with Goat-Anti-Mouse IgG (1 : 150, Molecular Probes) for 1 h, and nuclei were counterstained with 4,6-diamidino-2-phenylindole DAPI (1 : 1000, Molecular Probes) for 10 min and then rinsed with PBS. The fluorescent signal of cell nuclei and COL2 was visualized using a fluorescent microscope [36].

### 2.6. RNA Extraction and Quantitative Real-Time Polymerase Chain Reaction

Samples in each group were collected at the time point of 3 weeks of* in vitro* culture (*n* ≥ 3 per group). Total RNA was extracted from cell samples using TRIzol reagent (Invitrogen, USA) according to the manufacturer's instructions. cDNA was synthesized from total RNA using an Omniscript RT kit (Qiagen). The mRNA expression levels of SOX9, Col1a1, and Col2a1 were determined by real-time PCR using SYBR Premix EX Taq (Takara, Japan). The forward and reverse primer pairs were shown in Table 2. To normalize mRNA levels, the GAPDH housekeeping gene was used as an internal control [37].

### 2.7. Western Blotting

The expression levels of SOX9, COL2, and COL1 proteins from cell samples were analyzed as described previously [38]. Samples were lysed in RIPA lysis buffer at 4°C. Samples with equal protein concentration were subjected to SDS-PAGE and transferred to a PVDF membrane. Blots were blocked with 5% skim milk/TBS-Tween 20 for 1 h at room temperature and probed with primary antibodies: mouse anti-SOX9 (Santa Cruz Biotechnology, CA) and mouse anti-COL1 (Abcam, MA) and mouse anti-COL2 (Abcam, MA) and mouse anti-*β* actin (Abcam, MA) overnight at 4°C. Followed by which blots were washed with PBS-Tween 20 (0.1%) and incubated with horseradish peroxidase-conjugated secondary antibodies (1 : 1000) for 1 h at room temperature. Western blotting images are representative of *N* ≥ 3 images.

### 2.8. ELISA

The TGF-*β*1 concentration in the supernatant was determined by a human TGF-*β*1 ELISA kit (R&D Systems). BCA quantifying the total protein was conducted before ELISA; then the same protein quantity was loaded from different culture conditions. Absorbance was measured at a wavelength of 450 and 550 nm. The 450 nm values were subtracted from the 570 nm values for correction of the optical imperfections [39].

### 2.9. Cell Proliferation Assay

The cell viability was measured using a cell counting kit-8 (CCK-8) (Beyotime, Beijing, China) on days 0, 3, 6, 9, 12, and 15. Briefly, cells were seeded in 96-well plates at a density of 5000 cells/cm^2^ in DMEM/F-12 medium. CCK-8 solution (10 *μ*L) was added to each well. The cells were continually cultured for another 4 hours. During this period, viable cells could reduce the CCK-8 to formazan pigment, which was dissolved by 100 *μ*L culture medium. The number of viable cells was measured by recording the formazan pigment optical density at 450 nm by a microplate reader (Bio-Rad 380, USA) [5].

### 2.10. Statistical Analysis

The statistical significance was analyzed using SPSS statistical analytical software (ver. 18.0; IBM, USA). A Kruskal-Wallis test was used to assess differences among the groups. A* post hoc* test was performed along with a Mann-Whitney *U* test. *P* values less than 0.05 were considered to be statistically significant.

## 3. Results

### 3.1. Identification of hUCB-MSCs

**Figure 1:**
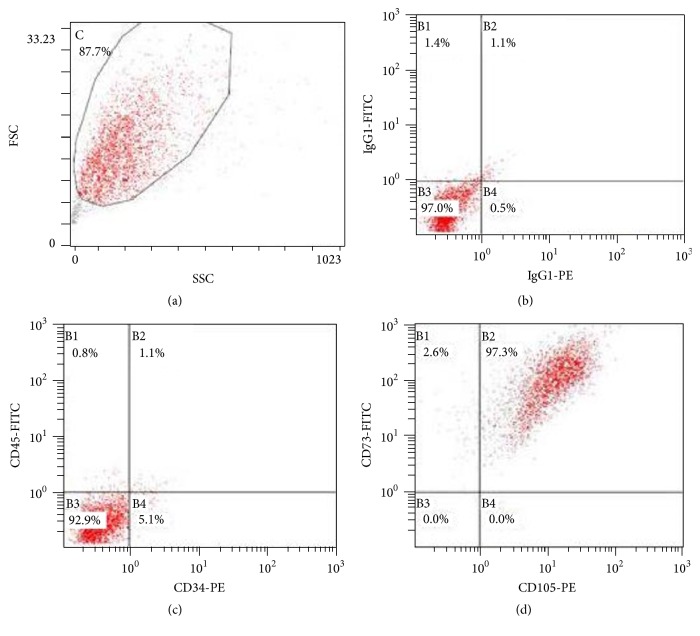
hUCB-MSC cell surface markers were characterized by flow cytometry. (a) Density plot showing the FSC to SSC. (b) IgG1-PE and IgG1-FITC were used as controls. (c) Cells expressing both CD34 and CD45 represented 1.1% of the population. (d) Cells expressing both CD105 and CD73 accounted for 97.3% of the population.

After the isolation of mesenchymal cells using the adherence criteria, the second passage of hUCB-MSCs was analyzed to confirm their identity. MSCs do not express CD34, CD45, CD117 (cKit), HLA class I, and HLA-DR antigens, whereas they are positive for CD13, CD29, CD44, CD73, CD90, CD105, and CD166 [40]. Surface markers considered positive for the mesenchymal cell lineage (CD73, CD105) and negative for the hematopoietic lineage (CD34, CD45) were used to characterize hUCB-MSCs. As shown in Figure 1, the flow cytometry analysis revealed that the isolated hUCB-MSC population had high expression of CD105 and CD73 and low expression of CD34 and CD45. Furthermore, these cells had acquired the fibroblastic morphology that is characteristic of mesenchymal stem cells, thus confirming the existence of mesenchymal stem cells in human umbilical cord blood. The proportion of stem cells met the identification criteria [41].

### 3.2. Immunofluorescence Staining of Collagen

**Figure 2:**
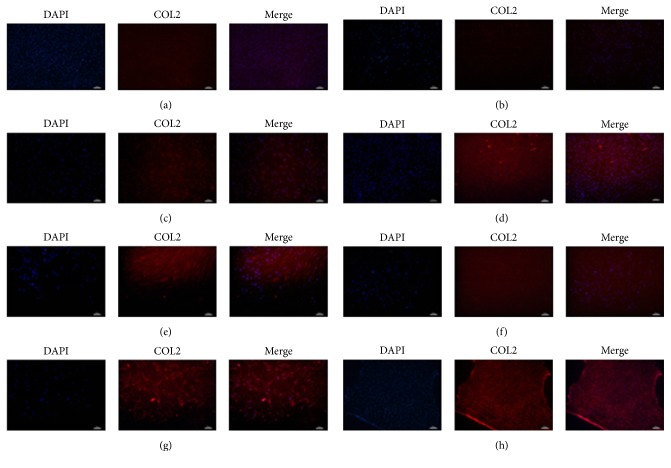
COL2 protein levels were characterized by immunofluorescence. Immunofluorescence staining of COL2 was shown in red. Nuclei were stained with DAPA (4′,6-diamidino-2-phenylindole, blue color). (a) hACs alone; (b) UCB-MSCs alone; (c) hUCB-MSCs cultured with growth factors; (d) direct coculture (1 : 1); (e) direct coculture (3 : 1); (f) direct coculture (5 : 1); (g) indirect coculture (UCB-MSCs); and (h) indirect coculture (hACs). Scale bar is 100 *μ*m. *N* = 5.

COL2 protein was minimally detectable by immunofluorescence staining in cultures containing only hUCB-MSCs, but significant levels of COL2 protein were detected in direct cell-cell contact coculture, indirect coculture, growth factor-supplemented hUCB-MSC culture, and hACs cultured alone. In all groups, COL2 protein was mainly distributed in the extracellular matrix (ECM). Coculture with direct cell-cell contact had stronger COL2 staining than the TGF*β*1-induced group. The TGF*β*1-induced group exhibited similar staining as compared to the indirect coculture group, and both stained more strongly than hUCB-MSCs cultured alone. The hUCB-MSCs cultured alone exhibited the weakest fluorescent signal among all the experimental groups, indicating that these progenitors do not undergo significant chondrogenesis in the absence of external signals. The hUCB-MSCs and hACs cocultured with direct cell-cell contact in a ratio of 3 : 1 stained stronger than the other ratios and conditions tested. However, all coculture groups with direct cell-cell contact showed signs of fibrosis. Immunofluorescence images of all groups stained for COL2 are shown in Figure 2.

### 3.3. Quantitative Analysis of mRNA Expression for Chondrocyte and Cartilage-Matrix-Related Genes

**Figure 3:**
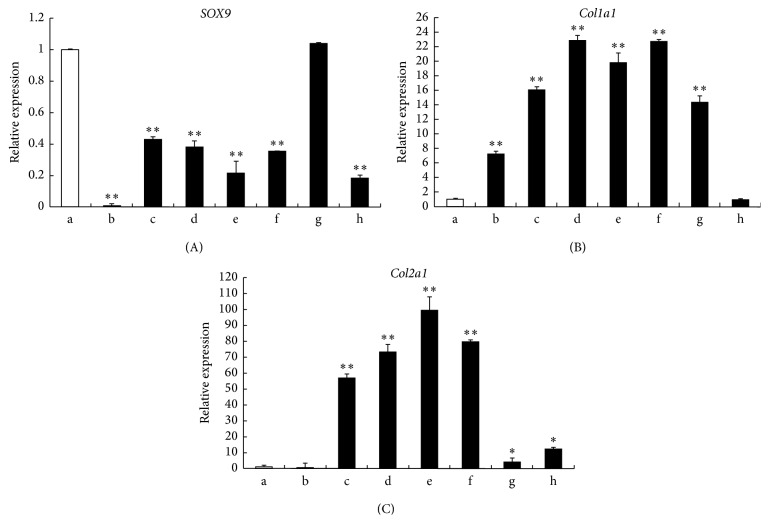
Gene expression was quantified with quantitative polymerase chain reaction. (A) SOX9, (B) Col1a1, and (C) Col2a1. Statistically significant differences were found by one-way ANOVA in all three genes (^*∗∗*^ *P* < 0.01). (a) hACs alone; (b) UCB-MSCs alone; (c) hUCB-MSCs cultured with growth factors; (d) direct coculture (1 : 1); (e) direct coculture (3 : 1); (f) direct coculture (5 : 1); (g) indirect coculture (UCB-MSCs); and (h) indirect coculture (hACs).

To evaluate chondrogenesis, the mRNA levels of several chondrocyte marker genes—*Col2a1*,* Col1a1*, and* SOX9*—were analyzed by quantitative real-time PCR after 3 weeks of culture* in vitro*. The hACs cultured alone had higher* SOX9* expression than hUCB-MSCs cultured alone or any of the cocultured groups (*P* < 0.05, Figure 3(A)).* Col2a1* and* Col1a1* expression in the direct cell-cell contact group was significantly upregulated relative to hACs alone (Figures 3(B) and 3(C)). Interesting, the* Col2a1* expression in indirect coculture did not show any significant change relative to hACs and the* Col2a1* expression was higher in the direct group than in the indirect coculture group. The hUCB-MSCs and hACs cocultured with direct cell-cell contact at the ratio of 3 : 1 had the highest* Col2a1* expression overall (Figure 3(C)).

### 3.4. Analysis of Chondrocyte and Cartilage-Matrix-Related Protein Production

**Figure 4:**
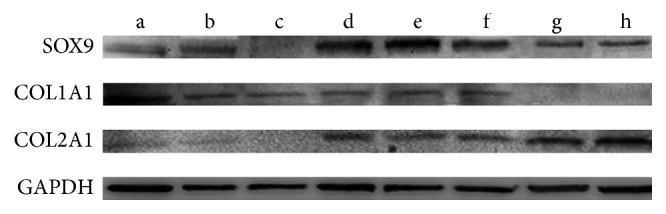
SOX9, COL2, and COL1 protein levels were detected by western blotting. (a) hACs alone; (b) UCB-MSCs alone; (c) hUCB-MSCs cultured with growth factors; (d) direct coculture (1 : 1); (e) direct coculture (3 : 1); (f) direct coculture (5 : 1); (g) indirect coculture (UCB-MSCs); and (h) indirect coculture (hACs).

To evaluate chondrogenesis in all groups, the production of chondrocyte marker proteins COL2, COL1, and SOX9 were analyzed by western blotting after 3 weeks of* in vitro* culture. SOX9 and COL2 proteins were upregulated in both direct cell-cell contact and indirect contact cocultures relative to monocultures. All three proteins were more abundant in cocultures with indirect cell-cell contact than in hUCB-MSC monoculture with supplemented growth factors, which actually had the lowest COL2 and SOX9 levels of any group. COL1 levels were highest in hAC monoculture, while indirect cell-cell contacted groups presented higher COL2 content than other groups (Figure 4).

### 3.5. ELISA Quantification of TGF-*β*1

**Figure 5:**
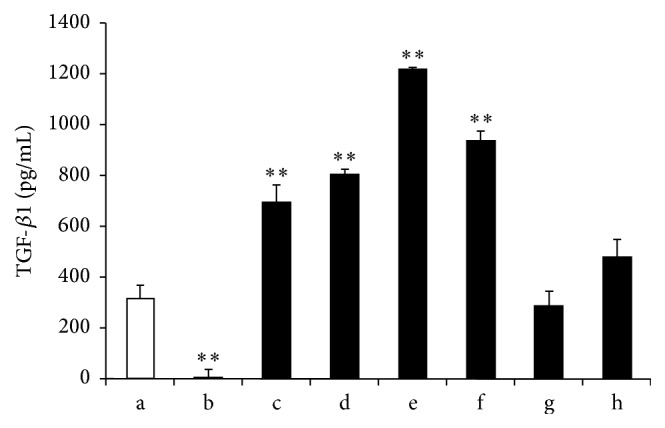
TGF-*β*1 secreted into the medium was measured using ELISA. Statistically significant differences were found by one-way ANOVA in TGF-*β*1 concentration. (a) hACs alone; (b) UCB-MSCs alone; (c) hUCB-MSCs cultured with growth factors; (d) direct coculture (1 : 1); (e) direct coculture (3 : 1); (f) direct coculture (5 : 1); (g) indirect coculture (UCB-MSCs); and (h) indirect coculture (hACs). ^*∗∗*^ *P* < 0.01.

The TGF-*β*1 protein expression level secreted into the medium was measured using ELISA after 3 weeks of* in vitro* culture. Secreted TGF-*β*1 was significantly upregulated in direct cell-cell contact coculture relative to hUCB-MSC monoculture supplemented with growth factors, which in turn was higher than in the indirect contact coculture (*P* < 0.01). TGF-*β*1 levels in indirect contact coculture were comparable to those in hACs cultured alone. The hUCB-MSC monoculture had the lowest TGF-*β*1 levels out of all groups. TGF-*β*1 concentration in the 3 : 1 direct contact coculture was highest among the groups tested (Figure 5).

### 3.6. CCK-8 Analysis of Cell Proliferation

**Figure 6:**
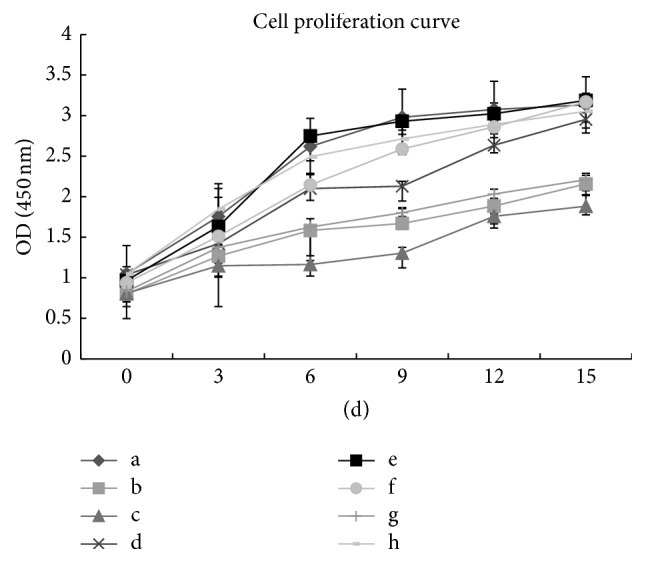
Analysis of cell proliferation. Statistically significant differences were found by one-way ANOVA. (a) hACs alone; (b) UCB-MSCs alone; (c) hUCB-MSCs cultured with growth factors; (d) direct coculture (1 : 1); (e) direct coculture (3 : 1); (f) direct coculture (5 : 1); (g) indirect coculture (UCB-MSCs); and (h) indirect coculture (hACs).

Total cellular proliferation in each of the different coculture conditions and with supplemental growth factors was quantified using a CCK-8 cell proliferation assay after 15 days of* in vitro* culture. The cell proliferation rates in the direct and indirect cell-cell contact coculture and hACs monoculture were similar. Cell proliferation was significantly stimulated in both coculture conditions and hAC monoculture relative to hUCB-MSC monoculture with growth factors, which had the lowest cell proliferation rate. Cellular proliferation in the direct cell-cell contact coculture at a ratio of 3 : 1 hUCB-MSCs to hACs was the greatest of all the tested conditions (Figure 6).

## 4. Discussion

Over the last two decades, there have been great improvements in the tissue engineering field for clinical repair of cartilage defects using autologous chondrocytes. However, the loss of phenotypic functions during chondrocyte expansion in monolayer culture has led to the search for an alternative cell source for cartilage tissue engineering. For example, BMSCs are currently undergoing trials for clinical practice in articular cartilage repair. However, BMSCs collection needs an invasive and painful process. hUCB-MSCs have numerous advantages over BMSCs, including convenient collection, better retention of their multipotency over several passages, reduced immunogenicity, absence of tumor cell contamination, and lower risk of latent virus and pathogenic microorganism transmission.

The hUCB-MSCs can be easily induced to differentiate into mature bone and cartilage cells by stimulation with growth factors, particularly with members of the TGF-*β* superfamily. Consistent with previous studies, chondrogenic induction by TGF-*β*1 and ITS could differentiate hUCB-MSCs into chondrocytes* in vitro*, although these cultures exhibited a fibrotic phenotype and limited cell proliferation rate after chondrogenic induction. In our study, COL1A1, a fibrosis marker, was significantly upregulated in hUCB-MSCs cultured with growth factors. The presence of type I collagen could impair the development of cartilage-specific matrix architecture and result in functional impairment. Therefore, our findings indicate that the commonly used chondrogenic induction medium for hUCB-MSCs is not optimal for cartilage regeneration and a better strategy to provide a more stringent control of hUCB-MSC chondrogenic differentiation can be achieved.

Recently, coculture systems for MSCs and chondrocytes were developed to enhance the chondrogenesis of MSCs [42]. Previous reports have demonstrated that coculture of hUCB-MSCs and rabbit chondrocytes could induce greater differentiation of hUCB-MSCs into chondrocytes [21]. However, the ratio of hUCB-MSCs to chondrocytes is an important element in coculture systems, and establishing the optimal ratio is crucial to successful coculturing and construction of cartilage tissue engineering [29]. But these methods barely exhibit the primary culture of hUCB-MSCs at low densities (≤10^4^ cells/cm^2^) for 1 month. We knew that the lower the cell density is, the more difficult it is to maintain the cells in culture. In this study, we performed the low density of hUCB-MSCs at 0.6 × 10^4^ cells/cm^2^. The density at 2.4 × 10^6^ cells/cm^2^ used by Zhang et al. was much higher than that (about 400-fold) adopted in our study. Our studies showed that low density coculture could induce hUCB-MSCs differentiation into chondrocytes, which resulted in high levels of COL2A1 mRNA and protein as well as increased COL1 expression. Thus, low density direct cell-cell contact could stimulate fibrocartilage-associated Col1a1 expression, which cannot meet the goal of hyaline cartilage for cartilage repair. However, compared with direct coculture, COL-1 expression was lower in the indirect coculture group. Interestingly, western blotting result showed that indirect coculture enhanced COL-2 expression while it inhibited COL-1 expression. Indirect coculture expresses type II collagen but not type I collagen, suggesting that indirect coculture has potential to induce hUCB-MSC differentiation into hyaline chondrocytes.

The mechanisms that contribute to enhancement of chondrogenesis in this hUCB-MSC and chondrocyte coculture system are unclear. Previous studies have shown the crucial role of the signaling cascade activated by TGF-*β*, which promotes the expression of genes specific to cartilage [23]. In several coculture experiments without direct cell-cell contact, TGF-*β* or other soluble factors alone contribute to cartilage-matrix formation and promote chondrogenic differentiation of MSCs [29]. Based on our* in vitro* studies, we found that hUCB-MSCs and hACs cocultured with direct cell-cell contact at ratio of 3 : 1 could secrete large amounts of TGF-*β*1. However, the relative importance of TGF-*β*1 and direct cell-cell contact in enhancing cartilage-matrix formation remains unclear. In this study, the concentration of TGF-*β*1 quantified by ELISA in culture media was generally much lower than that used for conventional chondrogenic induction and is most likely too low to induce efficient chondrogenic differentiation of MSCs on their own [24]. Although direct coculture secreted more TGF-*β*1 compared to indirect coculture, the Col1a1 expression was high, which means that the role of TGF-*β*1 may be also involved in the induced Col1a1 expression. And we found different expression levels of SOX9 between direct coculture and indirect coculture. Thus SOX9 may be involved in the induced chondrogenic differentiation of indirect coculture group. Additional studies have shown that SOX9 facilitates the COL2 expression, which promotes chondrogenesis [43], while another study found that SOX9 overexpression at high levels could exert an inhibitory effect on Col1a1 gene expression [44]. In this study, the SOX9 expression is higher in indirect coculture than in direct coculture or growth factor-inducing group. And the indirect coculture shows more COL2 expression. Thus, we presume that appropriate SOX9 in indirect coculture group could play an essential role in hUCB-MSC differentiation into chondrocytes and maintain chondrocytes phenotype. However, the exact role of SOX9 in coculture remains undefined, which needs further investigation.

In conclusion, indirect coculture at low density has potential to induce hUCB-MSC differentiation into hyaline chondrocytes. TGF-*β*1 may play a role in direct coculture that induces hUCB-MSCs into chondrocytes. SOX9 may be involved in the indirect coculture system that induces hUCB-MSCs into chondrocytes. Indirect coculture at low density could be a promising approach for repair of cartilage lesions.

## Figures and Tables

**Table 1:** The seeding cell number of human articular chondrocytes (hACs) and human umbilical cord blood-derived mesenchymal stem cell (hUCB-MSCs).

	Group	hUCB-MSCs (cells/cm^2^)	hACs (cells/cm^2^)
a	hACs	—	0.6 × 10^4^
b	hUCB-MSCs	0.6 × 10^4^	—
c	hUCB-MSCs cultured with growth factors	0.6 × 10^4^	—
d	Direct coculture (1 : 1)	0.3 × 10^4^	0.3 × 10^4^
e	Direct coculture (3 : 1)	0.45 × 10^4^	0.15 × 10^4^
f	Direct coculture (5 : 1)	0.5 × 10^4^	0.1 × 10^4^
g	Indirect coculture	0.6 × 10^4^	—
h	Indirect coculture	—	0.6 × 10^4^

**Table 2:** Primer sequences used for real-time PCR.

Genes	Forward primer (5′–3′)	Reverse primer (5′–3′)
SOX9	GACGTGCAAGCTGGGAAA	CGGCAGGTATTGGTCAAACTC
Col2a1	CGCCACGGTCCTACAATGTC	GTCACCTCTGGGTCCTTGTTCAC
Col1a1	GACATGTTCAGCTTTGTGGACCTC	GGGACCCTTAGGCCATTGTGTA
GAPDH	GGCACAGTCAAGGCTGAGAATG	ATGGTGGTGAAGACGCCAGTA

## References

[1] Makris E. A., Gomoll A. H., Malizos K. N., Hu J. C., Athanasiou K. A. (2015). Repair and tissue engineering techniques for articular cartilage. *Nature Reviews Rheumatology*.

[2] Kedage V. V., Sanghavi S. Y., Badnre A., Desai N. S. (2010). Autologous chondrocyte implantation (ACI): an innovative technique for articular cartilage defects. *Journal of Clinical Orthopaedics and Trauma*.

[3] Elima K., Vuorio E. (1989). Expression of mRNAs for collagens and other matrix components in dedifferentiating and redifferentiating human chondrocytes in culture. *FEBS Letters*.

[4] Yang Y.-H., Lee A. J., Barabino G. A. (2012). Coculture-driven mesenchymal stem cell-differentiated articular chondrocyte-like cells support neocartilage development. *Stem Cells Translational Medicine*.

[5] Kang N., Liu X., Guan Y. (2012). Effects of co-culturing BMSCs and auricular chondrocytes on the elastic modulus and hypertrophy of tissue engineered cartilage. *Biomaterials*.

[6] He X., Feng B., Huang C. (2015). Electrospun gelatin/polycaprolactone nanofibrous membranes combined with a coculture of bone marrow stromal cells and chondrocytes for cartilage engineering. *International Journal of Nanomedicine*.

[7] Buhrmann C., Mobasheri A., Matis U., Shakibaei M. (2010). Curcumin mediated suppression of nuclear factor-*κ*B promotes chondrogenic differentiation of mesenchymal stem cells in a high-density co-culture microenvironment. *Arthritis Research & Therapy*.

[8] Lindenmair A., Hatlapatka T., Kollwig G. (2012). Mesenchymal stem or stromal cells from amnion and umbilical cord tissue and their potential for clinical applications. *Cells*.

[9] Meretoja V. V., Dahlin R. L., Kasper F. K., Mikos A. G. (2012). Enhanced chondrogenesis in co-cultures with articular chondrocytes and mesenchymal stem cells. *Biomaterials*.

[10] Levorson E. J., Santoro M., Kurtis Kasper F., Mikos A. G. (2014). Direct and indirect co-culture of chondrocytes and mesenchymal stem cells for the generation of polymer/extracellular matrix hybrid constructs. *Acta Biomaterialia*.

[11] Rothenberg A. R., Ouyang L., Elisseeff J. H. (2011). Mesenchymal stem cell stimulation of tissue growth depends on differentiation state. *Stem Cells and Development*.

[12] Ghobadi F., Mehrabani D., Mehrabani G. (2015). Regenerative potential of endometrial stem cells: a mini review. *World Journal of Plastic Surgery*.

[13] Bhartiya D. (2015). Stem cells, progenitors & regenerative medicine: a retrospection. *Indian Journal of Medical Research*.

[14] Andrades J. A., Becerra J., Muñoz-Chápuli R. (2014). Stem cells therapy for regenerative medicine: principles of present and future practice. *Journal of Biomedical Science and Engineering*.

[15] Raoufi M., Tajik P., Dehghan M., Eini F., Barin A. (2010). Isolation and differentiation of mesenchymal stem cells from bovine umbilical cord blood. *Amsterdamer Beitrage zur Neueren Germanistik*.

[16] Pham P. V., Vu N. B., Pham V. M. (2014). Good manufacturing practice-compliant isolation and culture of human umbilical cord blood-derived mesenchymal stem cells. *Journal of Translational Medicine*.

[17] Cooper K., SenMajumdar A., Viswanathan C. (2010). Derivation, expansion and characterization of clinical grade mesenchymal stem cells from umbilical cord matrix using cord blood serum. *International Journal of Stem Cells*.

[18] Dahlin R. L., Ni M., Meretoja V. V., Kasper F. K., Mikos A. G. (2014). TGF-*β*3-induced chondrogenesis in co-cultures of chondrocytes and mesenchymal stem cells on biodegradable scaffolds. *Biomaterials*.

[19] Saraf A., Mikos A. G. (2006). Gene delivery strategies for cartilage tissue engineering. *Advanced Drug Delivery Reviews*.

[20] Fischer J., Dickhut A., Rickert M., Richter W. (2010). Human articular chondrocytes secrete parathyroid hormone-related protein and inhibit hypertrophy of mesenchymal stem cells in coculture during chondrogenesis. *Arthritis & Rheumatism*.

[21] Zheng P., Ju L., Jiang B. (2013). Chondrogenic differentiation of human umbilical cord blood-derived mesenchymal stem cells by co-culture with rabbit chondrocytes. *Molecular Medicine Reports*.

[22] Feng Z., Ying-Zhen W., Hai-Ning Z., Cheng-Yu L., Zong-Yao X. (2013). Differentiation of human umbilical cord blood derived-mesenchymal stem cells into chondrocytes induced by supernatants of human chondrocytes. *Journal of Clinical Rehabilitative Tissue Engineering Research*.

[23] De Mara C. S., Duarte A. S. S., Sartori A., Luzo A. C., Saad S. T. O., Coimbra I. B. (2010). Regulation of chondrogenesis by transforming growth factor-*β*
_3_ and insulin-like growth factor-1 from human mesenchymal umbilical cord blood cells. *Journal of Rheumatology*.

[24] Liu X., Sun H., Yan D. (2010). *In vivo* ectopic chondrogenesis of BMSCs directed by mature chondrocytes. *Biomaterials*.

[25] Koch T. G., Heerkens T., Thomsen P. D., Betts D. H. (2007). Isolation of mesenchymal stem cells from equine umbilical cord blood. *BMC Biotechnology*.

[26] Seo M.-S., Jeong Y.-H., Park J.-R. (2009). Isolation and characterization of canine umbilical cord blood-derived mesenchymal stem cells. *Journal of Veterinary Science*.

[27] Rosenzweig D. H., Matmati M., Khayat G., Chaudhry S., Hinz B., Quinn T. M. (2012). Culture of primary bovine chondrocytes on a continuously expanding surface inhibits dedifferentiation. *Tissue Engineering Part A*.

[28] Kang B.-J., Ryu H.-H., Park S. S. (2012). Comparing the osteogenic potential of canine mesenchymal stem cells derived from adipose tissues, bone marrow, umbilical cord blood, and Wharton's jelly for treating bone defects. *Journal of Veterinary Science*.

[29] Yang Y.-H., Lee A. J., Barabino G. A. (2012). Coculture-Driven Mesenchymal Stem Cell-differentiated articular chondrocyte-like cells support neocartilage development. *Stem Cells Translational Medicine*.

[30] Cooke M. E., Allon A. A., Cheng T. (2011). Structured three-dimensional co-culture of mesenchymal stem cells with chondrocytes promotes chondrogenic differentiation without hypertrophy. *Osteoarthritis and Cartilage*.

[31] Giovannini S., Diaz-Romero J., Aigner T., Heini P., Mainil-Varlet P., Nesic D. (2010). Micromass co-culture of human articular chondrocytes and human bone marrow mesenchymal stem cells to investigate stable neocartilage tissue formation in vitro. *European Cells and Materials*.

[32] Boeuf S., Richter W. (2010). Chondrogenesis of mesenchymal stem cells: role of tissue source and inducing factors. *Stem Cell Research & Therapy*.

[33] Wang M., Yang Y., Yang D. (2009). The immunomodulatory activity of human umbilical cord blood-derived mesenchymal stem cells in vitro. *Immunology*.

[34] Soleimani M., Khorsandi L., Atashi A., Nejaddehbashi F. (2014). Chondrogenic differentiation of human umbilical cord blood-derived unrestricted somatic stem cells on a 3D beta-tricalcium phosphate-alginate-gelatin scaffold. *Cell Journal*.

[35] Ma K., Titan A. L., Stafford M., Zheng C. H., Levenston M. E. (2012). Variations in chondrogenesis of human bone marrow-derived mesenchymal stem cells in fibrin/alginate blended hydrogels. *Acta Biomaterialia*.

[36] Wu L., Leijten J., van Blitterswijk C. A., Karperien M. (2013). Fibroblast growth factor-1 is a mesenchymal stromal cell-secreted factor stimulating proliferation of osteoarthritic chondrocytes in co-culture. *Stem Cells and Development*.

[37] Chang Q., Cui W.-D., Fan W.-M. (2011). Co-culture of chondrocytes and bone marrow mesenchymal stem cells in vitro enhances the expression of cartilaginous extracellular matrix components. *Brazilian Journal of Medical and Biological Research*.

[38] Kang J. S., Alliston T., Delston R., Derynck R. (2005). Repression of Runx2 function by TGF-*β* through recruitment of class II histone deacetylases by Smad3. *The EMBO Journal*.

[39] Li P., Wei X., Guan Y. (2014). MicroRNA-1 regulates chondrocyte phenotype by repressing histone deacetylase 4 during growth plate development. *The FASEB Journal*.

[40] Maurer M. H. (2011). Proteomic definitions of mesenchymal stem cells. *Stem Cells International*.

[41] Revencu T., Trifan V., Nacu L. (2013). Collection, isolation and characterization of the stem cells of umbilical cord blood. *Romanian Journal of Morphology and Embryology*.

[42] Yu D.-A., Han J., Kim B.-S. (2012). Stimulation of chondrogenic differentiation of mesenchymal stem cells. *International Journal of Stem Cells*.

[43] Kishi S., Abe H., Akiyama H. (2011). SOX9 protein induces a chondrogenic phenotype of mesangial cells and contributes to advanced diabetic nephropathy. *The Journal of Biological Chemistry*.

[44] Kypriotou M., Fossard-Demoor M., Chadjichristos C. (2003). SOX9 exerts a bifunctional effect on type II collagen gene (COL2A1) expression in chondrocytes depending on the differentiation state. *DNA and Cell Biology*.

